# The Male Gaze Explored: Ranking Thinness and Attractiveness of Female Body Shapes

**DOI:** 10.17505/jpor.2025.28095

**Published:** 2025-06-28

**Authors:** Revital Naor-Ziv, Yaarit Amram-Veitz, Joseph Glicksohn

**Affiliations:** 1 Department of Criminology, Bar-Ilan University, Israel; 2 The Leslie and Susan Gonda (Goldschmied) Multidisciplinary Brain Research Center, Bar-Ilan University, Israel

**Keywords:** body image, rank-ordering task, POSAC, male gaze, thinness, attractiveness, gestalt

## Abstract

The male gaze of the female body image in terms of thinness reveals its hierarchical structure: first torso, then legs, then arms. This is seen when in one task our male participants rank-ordered 8 composite images of a female body, derived from a Torso (thin vs. large) × Leg (thin/large vs. medium) × Arm (thin vs. large) design, from thinnest to largest. This primary focus on the torso is also critical in determining to what degree the female body image conforms to a desired hourglass shape. In a second task, the participants rank-ordered the same images from least attractive to most attractive. The rank-ordering of thinness was not readily predictive of the rank-ordering of attractiveness, and we found no clear end-structure underlying the female body image in terms of attractiveness. Nevertheless, in tracing the process of rating attractiveness, we can anchor the two ends of the series. The most attractive shapes were either Image 4 or Image 3, both instantiating a low waist-to-hip ratio (WHR). The first two choices for the least attractive shape were Image 1 (very thin) and Image 6 (very large). Great variability was found, however, in ranking the other body shapes, comprising composite images displaying an incompatibility of their various body parts. This results in a large number of paths for defining what attractiveness is for a particular male participant.

## Introduction

The body image (Grogan, 2017) is a *gestalt* (Werner, 1965), and the *male* gaze of the *female* body achieves “an overall or gestalt appreciation of the hourglass body shape incorporating a narrow waist and full breasts” (Dixson et al., 2011, p. 48). Using eye-tracking to trace the process of a man’s visual inspection of a female image, Dixson et al. (2011, p. 49) conclude that “men began to analyze essential components of the hourglass feminine shape, including the midriff and breasts, during the first 200 ms of viewing.” It is this hourglass shape which has, in turn, led to a focus on the waist-to-hip ratio (WHR)^1^ promoted by Singh (1993), and reported time and again in studies of male judgments of female attractiveness (Diekhoff et al., 2019; Dixson et al., 2011; Prantl & Gründl, 2011), with an ideal value approximating 0.7 (Del Zotto et al., 2020; Schützwohl, 2006; Singh, 1993). As Singh (1993, p. 304) puts it, the WHR “magnifies the sexual attractiveness of a woman who has shapely breasts and broad hips set against a narrow waist”. In Gestalt terms, this hourglass shape is a dominant and distinct figure on the background of the overall body, and is one that establishes a reference shape for comparing and contrasting other female body shapes (Gervais et al., 2013). Indeed, the hourglass figure can be contrasted with two other predominant female body shapes, the triangle and the rectangle (Makhanya et al., 2014), neither of which engage the male gaze. As a symmetric figure appearing on the background of the female body (Helson, 1925), the hourglass attracts the male gaze.

The bust-to-waist ratio (BWR), or its inverse, the waist-to-chest ratio (WCR), should also magnify this ‘sexual attractiveness’ (Diekhoff et al., 2019; Prantl & Gründl, 2011). When both BWR and WHR are taken into consideration, one can quantify the curvaceousness of the ‘hour-glass feminine shape’—for which WHR and 1/BWR are roughly equal (Shehi et al., 2012)^2^ —leading to some exaggerated ‘hour-glass shapes’, for both *Playboy* models (Pettijohn & Jungeberg, 2004, p. 1190) and *Marvel* superheroes (Burch & Johnsen, 2020, p. 122). It is this combination of a high BWR and a low WHR which results in the ‘supernormal’ female body image which especially attracts the male gaze (Burch & Johnsen, 2020; Marković & Bulut, 2017, 2023). On the other hand, as Furnham et al. (1998) emphasize, “large breasts consistently enhanced the attractiveness ratings of both slender and heavy figures‚ *so long as they had a low WHR*” (p. 321), while if the figure “had a high WHR‚ large breasts appeared to decrease the attractiveness ratings” (p. 322).

In this study, we return to a method that we had previously employed in investigating the perceived hierarchy of the body image (Naor-Ziv et al., 2020). This was to rank-order 8 composite images, derived from the familiar *Photographic Figure Rating Scale* (Swami et al., 2008), for which the torso, legs and arms were combined in a factorial design. In that study, our female participants were asked to rank-order the images from thinnest to largest, and an analysis of their individual profiles uncovered their hierarchical structure of the female body image. More specifically, in ordering the images from thinnest to largest, an individual profile is generated. Using partial-order scalogram analysis (POSAC), which maps these profiles into a 2D representational space, we reported that the space could be partitioned using parallel stripes perpendicular to a diagonal, and that when these are interpreted in terms of the image appearing in the third ordinal position within the profile, the hierarchical structure of the body image becomes apparent. The same method can also be employed to investigate the male gaze (Gervais et al., 2013) of the female body in terms of attractiveness. By decomposing the *gestalt* of the body image into its components, and then recombining these to create this series of female body shapes, we can see to what degree arm or leg width as ground can impact on the attractiveness of the central hour-glass figure.

In two previous studies in the literature, employing a hefty factorial recombination of body parts (Gründl et al., 2009, 243 variations; Smith et al., 2007, 625 variations), WHR and BHR were clear predictors of attractiveness, but so were other components, such as thigh width (see also Garza et al., 2016, p. 13). Nevertheless, it has been reported that neither the arms nor the legs seem to be relevant in judging attractiveness (Cundall & Guo, 2017, p. 9; Rodway et al., 2018, p. 354; Smith et al., 2007, p. 949). If so, then their impact on the central hour-glass *figure* should be minimal. Indeed, time and again it has been reported that it is the midriff area (Garza et al., 2016; Rozmus-Wrzesinska & Pawlowski, 2005) together with the hip area (Bovet et al., 2016) and the breasts (Furnham et al., 1990) which attract the male gaze.

Furthermore, not only can we focus on structure—or end structure—of this rank-ordering procedure, we can also trace the process of a man’s visual inspection of a female image, by looking at the consecutive choice-by-choice ratings of thinness, on the one hand, and of attractiveness, on the other. This emphasis on *process* rather than end-product or structure is of prime importance (Werner, 1937), because a study of the male gaze must surely consider the process driven by “a focus on women’s sexual body parts, not by a focus on non-sexualised body parts such as arms….” (Bernard et al., 2018, p. 111). However, the incompatibility of these body parts in our composite images might very well impact on that process. That is to say, in our composite images the various body parts do not preserve proportionality, as in other studies (e.g., Furnham et al., 2006). Nor is it the case that only one body part at a time is exaggerated here, as done in other studies (e.g., Wiggins et al., 1968). Hence in the present study, thin arms can appear together with large legs, for example, providing incompatible information regarding body weight. Given that “there may be many routes to being assigned a particular attractiveness level” (Smith et al., 2007, p. 950), and that “it is possible that judgements of attractiveness are driven by a more holistic appraisal of body shape” (Smith et al., 2007, p. 929), it is important to establish these different paths. Indeed, Pokrywka et al. (2006, p. 1694) suggested that “we should pay attention not only to the thinness and curvaceousness of the body (hour-glass), but also to slender legs.”

The three questions studied are as follows: (1) Can we replicate our 2D POSAC solution for the rank-ordered size of these same female body images, now using male participants, given that the male view of the ideal female body size differs from that of the female view (Diekhoff et al., 2019; Johnson & Engeln, 2021)? (2) What will be the lawfulness for rank-ordered attractiveness? (3) What is the relationship between rank-ordered size and rank-ordered attractiveness, given that it is not clear that thinner female body images will invariantly be viewed as being more attractive (Swami & Tovée, 2012; Wilson et al., 2005)?

## Method

### Participants

Sixty-four male undergraduate students studying at a regional college participated in this study. They ranged in age between 21 and 67 years (Mean age = 30.2, *SD* = 8.4). All participants provided informed consent, and they were assured of the confidentiality of their data; the study was approved by the university ethics board. The study was run by the second author in a one-to-one individual session in one of the classrooms. No remuneration was given, and participation was strictly voluntary.

### Rank Ordering Tasks

**Figure 1:**
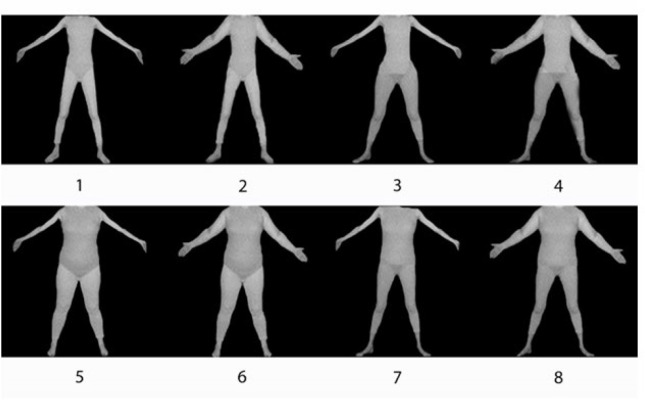
The 8 images used for the rank-ordering tasks. Image 1 (TTT: thin torso, thin leg, thin arm); Image 2 (TTL: thin torso, thin leg, large arm); Image 3 (TMT: thin torso, medium leg, thin arm); Image 4 (TML: thin torso, medium leg, large arm); Image 5 (LLT: large torso, large leg, thin arm); Image 6 (LLL: large torso, large leg, large arm); Image 7 (LMT: large torso, medium leg, thin arm); and Image 8 (LML: large torso, medium leg, large arm).

We employed images taken from the familiar *Photographic Figure Rating Scale* (PFRS; Swami et al., 2008)—the images being calibrated with actual BMI (Body Mass Index) values, as reported by Swami et al. (2008). Image 2 (BMI = 14.72), Image 5 (BMI = 20.33), and Image 8 (BMI = 29.26) of the *PFRS* were manipulated using Adobe Photoshop, set to 600 pixels per square inch for all stages. These images, which are equidistant on the *PFRS*, were chosen to be representative of thin, medium, and large body shapes. These were divided into torso, legs and arms, and these body parts were saved as individual images (130 mm × 130 mm). From these we could construct eight composite images, based on the respective widths of a Torso (thin vs. large) × Leg (thin/large vs. medium) × Arm (thin vs. large) design. These images appear in Figure 1. Image 1 (thin torso, thin leg, thin arm; TTT) is one of the original figures appearing in the PFRS, as is Image 6 (large torso, large leg, large arm; LLL). All other images were constructed to provide the different combinations required by the factorial design, as described above. The images were printed on separate cards.

In one task, our participants were asked to rank-order these from the thinnest to the largest body shape, as in the previous study (Naor-Ziv et al., 2020). In the second task, our participants were asked to rank-order these from the least attractive to the most attractive.

### Morphometrics

A grid was prepared using the drawing tool of PowerPoint, delineating horizontally (widthwise) bust width, waist width and hip width, and was superimposed on each of the 8 imaages employed in this study, within PowerPoint, adjusting relative distances within each image, and then each such image was saved as TIFF. Each image was subsequently imported into Image, version 1.63 (open-source software, NIH, Bethesda), wherein each of the 3 distances was measured along the superimposed grid. We subsequently computed both the waist-to-hip ratio (WHR) and the bust-to-waist ratio (BWR) as our two dimensionless morphometric measures

### Procedure

Each participant was asked to rank-order the series of 8 female body images, in one task from thinnest to largest, or from largest to thinnest, and in a second task, from least attractive to most attractive, or from most attractive to least attractive. The second author presented these cards to the participant in a random order (achieved by shuffling the cards before the participant). The two tasks were counterbalanced, as was the direction of rank-ordering. The order of the cards presented to the participant was photographed for reference. This was then compared to the order established by the participant, which was also photographed.

### Data Analysis

Given the nature of our rank-ordering tasks, the individual profile generated necessarily presents correlated choices. To alleviate this situation, in analyzing the profile data for the rank-ordering of thinness, we look at only the first five rankings and employ a partial order scalogram analysis by coordinates (POSAC) procedure, as described by Shye (2007), which maps these profiles into a 2D representational space. POSAC extends the familiar 1D Guttmann scale to a 2D representational space (Shye, 2014), preserving partial order among the various individual profles. The analysis was run using the POSAC module of the Hebrew University Data Analysis Package (HUDAP; Guttman & Greenbaum, 1998). This analysis portrays the structure underlying these rank orderings. We supplemented this analysis with cluster analysis, using both Stata and ROPstat (Vargha et al., 2015), following the implementation of Ward's method (with the squared Euclidean distance being the distance measure between clusters). We note that even though the data are clearly ordinal in nature, cluster analysis can still be employed here (Mooi et al., 2018, p. 337). In order to trace the process of rank-ordering the images, we computed the sequential choice-by-choice ratings of thinness and attractiveness, as will be described in the Results.

## Results

### Rank-Ordering of Thinness

**Figure 2:**
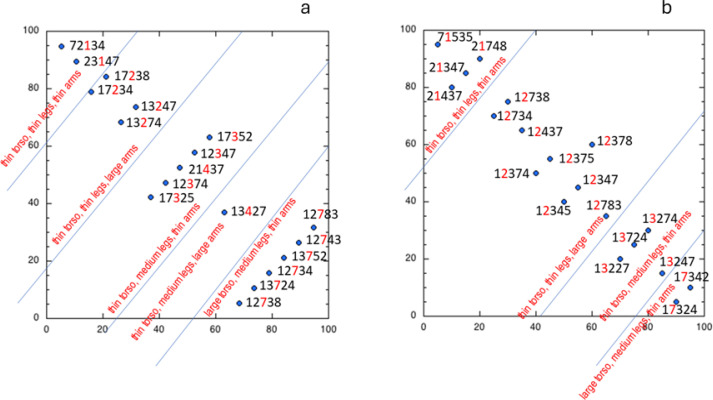
POSAC (partial-order scalogram analysis) space in which the various profiles can be mapped, with these appearing along one diagonal. a: the data of our female participants, as previously reported (Naor-Ziv et al., 2020). b: the data for our male participants.

Figure 2a presents the 2D POSAC space for the data of our female participants, as previously reported (Naor-Ziv et al., 2020), which can now be compared to Figure 2b, presenting the data for our male participants. Note the following: (1) the profiles again lie on the top-left to bottom-right diagonal, indicating a unidimensional mapping; (2) a lawful partitioning can be seen when focusing on the images appearing in the third ordinal position for the females, and in the second ordinal position for the males; (3) the space can be partitioned using parallel stripes perpendicular to this diagonal, indicating the hierarchical structure of the body image underlying these rankings of thinness.

The hierarchical structure of the body image that we uncover using POSAC is: first torso, then legs, then arms. This hierarchy becomes most effective for the third ordinal decision made by the female participants (Naor-Ziv et al., 2020), and for the second ordinal position made by the male participants, in rank-ordering the images, indicating that the male gaze is faster at determining this hierarchy.

**Figure 3:**
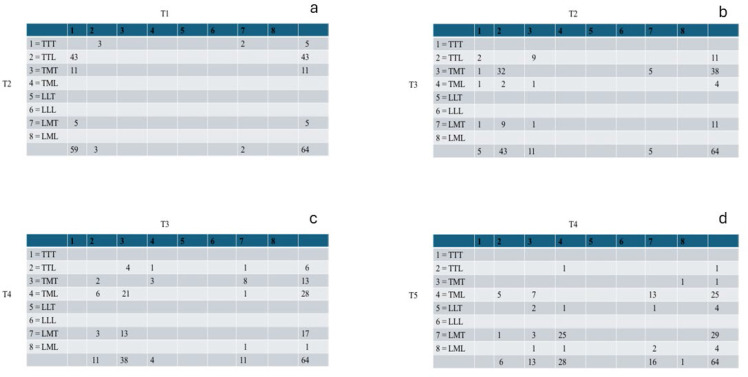
a: first ranking of thinness (T1) by the second ranking of thinness (T2). b: second ranking of thinness (T2) by the third ranking of thinness (T3). c: third ranking of thinness (T3) by the fourth ranking of thinness (T4). d: fourth ranking of thinness (T4) by the fifth ranking of thinness (T5).

Turning to the process of rank-ordering the images according to thinness, we looked at the consecutive choice-by-choice ratings of thinness, the first four stages being summarized in Figure 3. Figure 3a presents the cross-tabulation of the first ranking of thinness (T1) by the second ranking of thinness (T2). Image 1 (TTT) was predominantly chosen (59/64) as the thinnest body shape, usually followed (43/64) by Image 2 (TTL). The third image chosen is usually Image 3 (TMT; 38/64; Figure 3b); the fourth image chosen is usually Image 4 (TML; 28/64; Figure 3c), usually followed by Image 7 (LMT; 29/64; Figure 3d) as the fifth image. The sixth image chosen is usually either Image 8 (LML; 23/64) or Image 5 (LLT; 28/64), each of which is subsequently followed by the other image in seventh rank-order, namely Image 5 (22/64) or Image 8 (21/64). The last ranking image in the sequence is then usually Image 6 (40/64).

**Figure 4:**
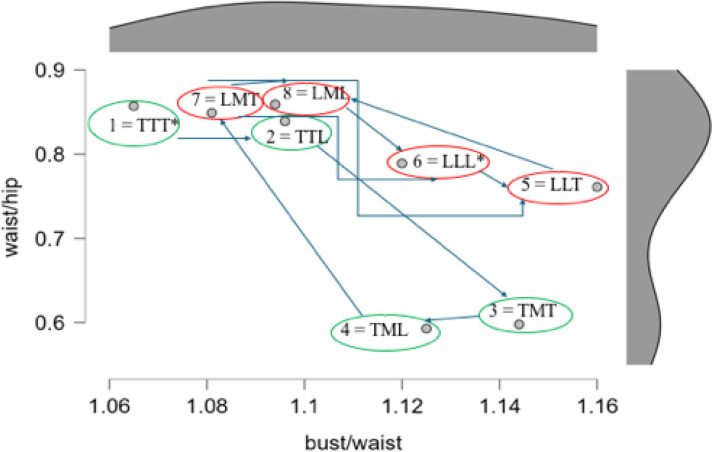
Sequential unfolding of this rank-ordering process, mapped onto the plane defined by the waist-to-hip ratio (WHR) and the bust-to-waist ratio (BWR). Respective distributions appear in the margins.

Figure 4 presents this sequential unfolding of this rank-ordering process, mapped onto the plane defined by the waist-to-hip ratio (WHR) and the bust-to-waist ratio (BWR). Note that the first four choices all have a common thin torso, modified by a thin or medium size leg, and then by a thin or large size arm. The next four choices all have a common large torso, modified by a medium or large size leg, and then by a thin or large arm.

### Rank-Ordering of Attractiveness

We find no orderly partitioning of a 2D POSAC space for attractiveness: A total of 62 different profiles were found, sample size being 64. Inspection of these profiles suggested two major clusters for defining the least attractive image: Image 1 (TTT) and Image 6 (LLL). The most attractive image was either Image 3 (TMT) or Image 4 (TML). The clustering solution provided by Stata, implementing Ward’s (using the squared Euclidean distance metric) uncovered two large clusters: one cluster with a total *n* = 34; and a second cluster with a total *n* = 30.

A subsequent analysis was conducted using the clustering module of ROPstat and employing Ward’s method, followed by a *k*-clustering tweaking of that cluster solution, for *k* ranging between 2 and 4. For *k* = 2, we find cluster 1 (*n* = 34) starting off with the lowest rankings (thin torso and thin legs), and cluster 2 (*n* = 30) starting off with the highest values (large torso and large legs). The summary statistics here are as follows: Explained Error Sum of Squares (ESS) proportion = 25%, which is far from being high (Malmberg & Little, 2007, p. 744); and point-biserial correlation (PB) = .48. Moving now to *k* = 4, ESS = 47%, which again is not high; and PB = .50, which is slightly higher than the previous value. In agreement with an anonymous reviewer’s comments, the existence of these clusters is tenuous, at best. Evidently, as also indicated in the analysis run using POSAC, there is much individual variability here in rank-ordering attractiveness. Nevertheless, the least attractive (cluster 1—thin torso and thin legs; or cluster 2—large torso and large legs) and the most attractive images (thin torso and medium legs) can be established.

**Figure 5:**
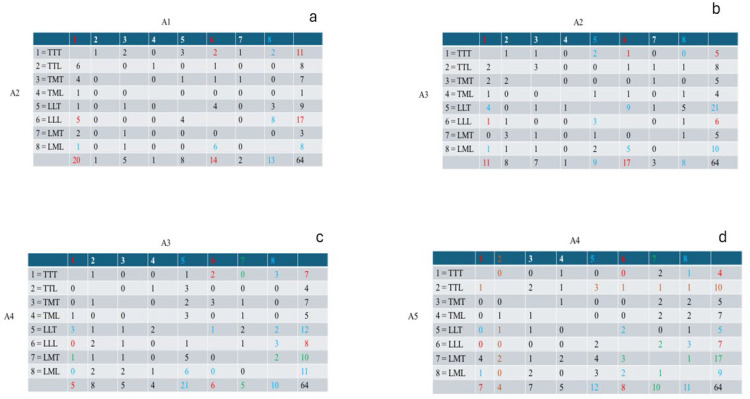
a: first ranking of attractiveness (A1) by the second ranking of attractiveness (A2). b: second ranking of attractiveness (A2) by the third ranking of attractiveness (A3). c: third ranking of attractiveness (A3) by the fourth ranking of attractiveness (A4). d: fourth ranking of attractiveness (A4) by the fifth ranking of attractiveness (A5).

Turning to the *process* of rank-ordering the images according to attractiveness, we looked at the consecutive choice-by-choice ratings of attractiveness, the first four stages being summarized in Figure 5. Figure 5a presents the cross-tabulation of the least attractive body shape (A1) by the second ranking of attractiveness (A2). Image 1 (TTT) was predominantly chosen (20/64) as the least attractive body shape, usually followed (17/64) by Image 6 (LLL). The third or fourth image chosen is usually Image 5 (LLT; 21/64; Figure 5c) or Image 8 (LML; 11/64; Figure 5c), usually followed by Image 7 (LMT; 17/64) or Image 2 (LML; 10/64), and subsequently followed by the most attractive body shapes, Image 4 (19/64) and Image 3 (20/64).

**Figure 6:**
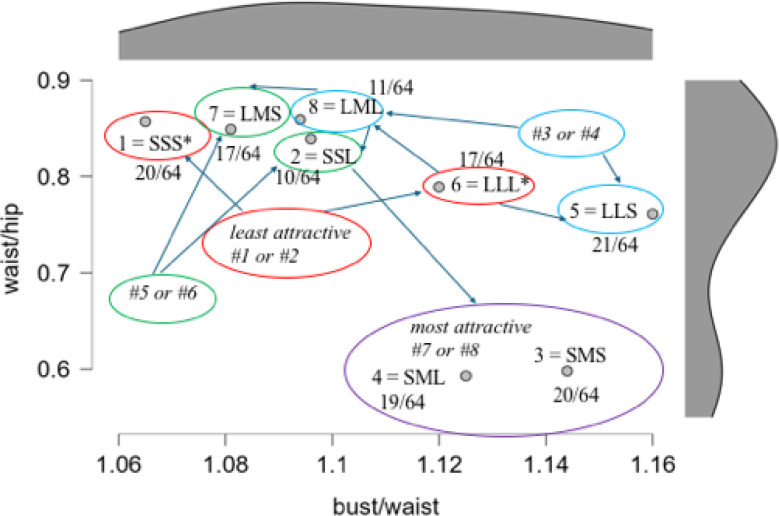
Sequential unfolding of this rank-ordering process, mapped onto the plane defined by the waist-to-hip ratio (WHR) and the bust-to-waist ratio (BWR). Respective distributions appear in the margins.

Figure 6 presents this sequential unfolding of the rank-ordering process, mapped onto the same plane defined by the waist-to-hip ratio (WHR) and the bust-to-waist ratio (BWR). Note that the WHR range is the same as that employed by Del Zotto et al. (2020), while the BWR range is much less than that found by Makhanya et al. (2014), but within the range found by Shehi et al. (2012). The first two choices for the least attractive shape are Image 1 (TTT; 20/64) and Image 6 (LLL; 17/64), which are markedly either very thin or very large. Choices #3 and #4 are then usually Image 8 (LML; 11/64) and Image 5 (LLT; 21/64), both of which modify slightly the previous very large figure. Choices #5 and #6 are then usually Image 7 (LMT; 17/64) and Image 2 (TTL; 10/64). Finally, the most attractive images are ranked #7 and #8, these usually being Image 4 (TML; 19/64) and Image 3 (TMT; 20/64), both having small WHR.

## Discussion

The hierarchical structure of the female body image in terms of thinness is first torso, then legs, then arms (Naor-Ziv et al., 2020). Our male participants were able to uncover this hierarchy already in their second ordinal ranking. Turning to their process of rank-ordering the images according to thinness, their first 4 choices (thinnest to less thin) all have a thin torso, then thin or medium legs, then thin or large arm. Their next 4 choices all have a large torso, then medium or large legs, then thin or large arms. Thus, the process of rank-ordering the images is in complete alignment with the end-structure. Note that this primary focus on the torso is also critical in determining to what degree the female body image conforms to a desired hour-glass shape (Dixson et al., 2010).

This rank-ordering of thinness is, however, not readily predictive of the rank-ordering of attractiveness. In fact, we found no clear end-structure underlying the female body image in terms of attractiveness. Instead, we found 62 distinct profiles in our sample of 64 male participants. Nevertheless, their process of rank-ordering the images did reveal a clear aversion for those images which were markedly either very thin, which comes as no surprise (Diekhoff et al., 2019, p. 8; Johnson & Engeln, 2021, p. 304), or very large, as expected (Lassek & Gaulin, 2016, p. 12). That the female body shape with a small WHR approximating 0.7 is found to be most attractive is also a consensual finding (Prantl & Gründl, 2011; Singh, 1993; Streeter & McBurney, 2003). Hence, in tracing the process of rating attractiveness, we can anchor the two ends of the series. The most attractive shapes were either Image 4 or Image 3, both instantiating low WHR/high BHR, in line with the results reported by Brooks et al. (2010, p. 2245), that “BWR was a significant predictor of attractiveness … but not quite as good as WHR”.

Great variability is found, however, in ranking the other body shapes, comprising composite images displaying an incompatibility of their various body parts. While the female body shapes usually employed in the literature preserve the “thickness of the arms and legs” (Furnham et al., 2006, pp. 452-453), our images displayed an incompatibility here. Consider Image 7 and Image 2, both of which were usually ranked in the fifth and sixth places (i.e., as being quite attractive). Image 7 might be rated as being quite attractive because a large torso is coupled with medium legs, where a medium-thigh width “can be considered beautiful” (Manzaneda Cipriani et al., 2022, p. 2). Image 2 might be rated as being quite attractive because a thin torso is coupled with thin legs, suggesting an “underweight female figure” which may also be considered to be attractive (Pettijohn & Jungeberg, 2004, p. 1193; Puhl & Boland, 2001, p. 42), but not also with thin arms, because “Thin was ideal; too thin was not” (Diekhoff et al., 2019, p. 8). This incompatibility of the various body parts results in incompatible information regarding body weight. In essence then, WHR and BMI, the two competing predictors of attractiveness (Tovée & Cornelissen, 2001; Tovée et al., 1999; Wilson et al., 2005), have been dissociated in this study. This suggests a promising avenue for future research. A second promising area results from considering the fact that even though the composite figures are far from being well-formed gestalten, they do suggest that attractive images may not adhere to strict proportionality. In fact, the various body parts interact with each other, such that preference is determined by their gestalt (Lavrakas, 1975; Wiggins & Wiggins, 1969). Furthermore, individual differences in preference for these composites or configurations is clearly apparent: Wiggins and Wiggins (1969) reported a subdivision of their sample into five groups showing differential preference for different body shapes. The results of the present study can certainly confirm this great variability in the end-structure (profile) produced.

Potential limitations of the present study, raised by Puhl and Boland (2001, p. 29) in their criticism of Singh’s (1993) rank-ordering studies, are as follows: (1) this type of design is susceptible to the demand characteristics of the task—to which we respond that given our composite images, it is far from clear what image is expected to be attractive; (2) that rank-ordering is not as powerful (statistically) as using rating scales—to which we respond that rank-ordering is a powerful method, when coupled with the use of POSAC (Naor-Ziv et al., 2020; Shye, 2007), to uncover the end-structure, if this exists (for thinness, it does; for attractiveness, it doesn’t). Another limitation lies in the fact that in selecting composite images for use in a rank-ordering task, one has to forego the use of an extensive crossing of the various values for each dimension, in order to produce a task that is not too taxing for the participant. In this study, we employed 8 different images, in line with a Torso (thin vs. large) × Leg (thin/large vs. medium) × Arm (thin vs. large) design. Had we insisted on having 3 width values for each body part (thin, medium, large), this would have resulted in 27 different images—which would clearly impede on the feasibility of the rank-ordering task. Other authors have had to make similar compromises when investigating the attractiveness of body shapes (e.g., Furnham et al., 1990; Wiggins et al., 1968). An anonymous reviewer has suggested that our sample size of 64 is another limitation—our data not allowing for generalization, and not likely to replicate. In reply, we note that in our previous study in this domain (Naor-Ziv et al., 2020), the sample size there was even smaller (*n* = 44), though samples of such size do appear in the literature on the body image (Thaler et al., 2018), and in the literature employing a rank-ordering task (Baird & Brier, 1981; Böhm & Pfister, 1996). Regarding replication, the present study was able to replicate the previous study’s findings regarding the rank-ordering of thinness.

Various ‘odd’ composite figures can be rated as being quite attractive, resulting in a large number of paths for defining what attractiveness is for a particular male participant.^3^ Even low WHR is not uniformally preferred by male participants as indicating the most attractive body shape (Kościński, 2014). As Smith et al. (2007, p. 950) suggest,

When observers are in attractiveness preference mode, one possibility is simply that “attractiveness space” has multiple maxima, i.e., different configurations of physical features can produce the same level of attractiveness…. For example, a body with a higher BMI may be compensated for by a more curvaceous WHR and WCR, and this configuration might be rated as attractive as a body with a more attractive BMI and less curvaceous WHR and WCR. Thus, there may be many routes to being assigned a particular attractiveness level….

It would seem that the male gaze is quite variable, and that beauty is indeed in the eye of the beholder.

## Data Availability

The data reported here will be made available, following such a request to the corresponding author.

## Footnotes

1 The WHR is an indicator of obesity and a predictor of various health problems, such as type 2 diabetes (high WHR; Jaeschke et al., 2015). At the same time, WHR is an indicator of fertility and good health (low WHR; Tovée et al., 1999), and is negatively correlated with attractiveness (Rozmus-Wrzesinska & Pawlowski, 2005).

2 Shehi et al. (2012, p. 79) incorrectly write BWR, but it is clear that it is the inverse value which should be indicated.

3 Following an anonymous reviewer’s suggestion, we comment here on whether the male rater’s own body shape could have influenced his rank-ordering of attractiveness. Not having data on their morphometric measures, such as the male waist-to-chest ratio (WCR), or self-reported BMI, we can only speculate, based on the findings of others. Two studies stand out. Price et al. (2013, p. 8) reported that “in males high sociosexuality and self-perceived attractiveness were associated with greater strength of preference for attractive traits when judging female bodies”, but also that their own WCR was not a significant predictor of female body-shape attractiveness. Wang et al. (2018), who, as in the present study, reported “tremendous variability in the ratings of attractiveness given to any particular image” (p. 523), tended to downplay the role that self-reported BMI of their male raters might have on their attractiveness ratings for female body shapes (i.e., whether male raters with higher BMI would favour images of women also having high BMI). Nevertheless, they did report (p. 527) that “Caucasian males with higher BMIs on average rated images of thinner subjects slightly lower and images of individuals with obesity slightly higher”. While this suggests that further research is required on this interesting topic, we would also stress that, as Rosinger and Puts (2018) suggest, “it’s complicated”.

## Action editor

Lars-Gunnar Lundh served as action editor for this article.

## Open access

This article is distributed under the terms of the Creative Commons Attribution 4.0 International License (http://creativecommons.org/licenses/by/4.0/), which permits unrestricted use, distribution, and reproduction in any medium, provided you give appropriate credit to the original author(s) and the source, provide a link to the Creative Commons license, and indicate if changes were made.
